# Hand in Glove: Health Equity and Learning Health Systems

**DOI:** 10.1002/lrh2.70078

**Published:** 2026-04-16

**Authors:** Susan Lacey Bryant, Gerald J. Perry, Terrie R. Wheeler

**Affiliations:** ^1^ Visiting Professor in Knowledge and Information Mobilisation Manchester Metropolitan University Manchester UK; ^2^ Associate Dean Emeritus University of Arizona Libraries Tucson Arizona USA; ^3^ Samuel J. Wood Library and C.V. Starr Biomedical Information Center, Weill Cornell Medicine New York New York USA

1

In 2024, members of the Health Equity and Access to Learning (HEAL) Workgroup of the Mobilizing Computable Biomedical Knowledge—North America Chapter (MCBK‐NA) [https://mobilizecbk.med.umich.edu/workgroups/health‐equity‐and‐access‐to‐learning, https://mobilizecbk.med.umich.edu/] published a call to action directed to the biomedical library and information sciences community [1]. The call encouraged librarians and information professionals to work in partnership with the biomedical informatics community and others to advance the work of health equity as a key component of the MCBK movement.

MCBK is an interdisciplinary, international community that is working to ensure that biomedical knowledge as expressed in computable forms is findable, accessible, interoperable, reusable (FAIR) and equitable. The effort is rooted in a core belief that health‐related decisions should be informed by the best available knowledge. As a community, we believe that machine‐readable health knowledge is increasingly a critical component of the healthcare enterprise. With such technological advancements comes tremendous opportunities for solutions at scale as well as more precise applications of evidence‐based knowledge to improve the health and care of individuals and entire communities [https://mobilizecbk.med.umich.edu/].

This Special Issue on *Health Equity and Learning Health Systems* is intended as the next step in the HEAL Workgroup's advocacy through scholarship effort.

As Co‐editors for this issue, all librarians by profession, we believe that:

- Learning health systems (LHSs) are a means for advancing health equity in an inclusive and systematic manner;
- Creating a bridge between knowledge generation and its implementation is essential to learning and continuous improvement within health systems [2]—librarians and other information professionals are essential contributors to these efforts,
- This work is part of a larger international movement with immediate roots in the larger MCBK effort [3]; and
- Work being done in the National Health Service (NHS) in England led by the national NHS Knowledge and Library Services team to ensure equitable access to knowledge resources and services for staff can inform approaches to improve health equity internationally [4].

We are committed to the goal of health equity because we recognize that we live in a world increasingly challenged to provide equitable health care to all people in need. There is a tendency for leading edge biomedical technologies and resources to be available only to those who can afford them, thus increasing disparities that are already at unacceptable levels. Despite the recent politicization of the concept of health equity in the United States and widening inequalities in life expectancy between regions in England over the past 15 years [5], from an ethical perspective there can be no denying that one's postal or zip code should not define one's access to quality health care.

We believe that the approaches taken by LHSs have the potential to amplify health equity efforts. LHSs encourage and support the full engagement of patients, providers, payers, and information and implementation professionals to improve health and health care, employing equitable methods in pursuit of equitable outcomes [6]. This Special Issue therefore focuses on health equity as both a goal and driver, leveraging the benefits of LHSs. We invited authors to share their theories, research, and/or programmatic experiences connecting LHSs to efforts to build a fairer, healthier world. A special invitation was extended to individuals in health information professions (librarians, knowledge specialists, informaticists, and others) who engage with diverse communities to advance the goal of health equity. The perspectives included herein represent contributions from authors situated in the United States (US) and the United Kingdom (UK), acknowledging that health equity issues are international and exist regardless of national approaches to health care provisioning.

Conceptions of how health equity is to be defined are diverse and have evolved over time. Several contributors to this Special Issue reference the work of University of California, San Francisco researcher and scholar Paula Braveman, who with a team of colleagues developed a definition for the Robert Wood Johnson Foundation entitled, *What is Health Equity? And What Difference Does a Definition Make?* published in 2017: “Health equity means that everyone has a fair and just opportunity to be as healthy as possible. This requires removing obstacles to health such as poverty, discrimination, and their consequences, including powerlessness and lack of access to good jobs with fair pay, quality education and housing, safe environments, and health care” [7].

Our understanding of the concept additionally draws on the scholarship around social determinants of health and health disparities [8, 9, 10]. Our work is also informed by the recent experience of the COVID‐19 pandemic, which highlighted uncomfortable truths about persistent health inequities in societies across the globe [11, 12]. As librarians, we also recognize the challenges of access to quality health information services and resources, whether in our communities or in our institutions and systems, local or national [13]. These systemic challenges inform our conviction that it is important for LHSs to be explicit in providing means and mechanisms for enhancing the application of health knowledge in the pursuit of equitable health care.

Our working definition of LHSs is found in the groundbreaking US Institute of Medicine's 2013 work, *Best Care at Lower Cost: the Path to Continuously Learning Health Care in America*: “A system in which science, informatics, incentives, and culture are aligned for continuous improvement and innovation, with best practices seamlessly embedded in the care process, patients and families as active participants in all elements, and new knowledge is captured as an integral by‐product of the care experience” [14].

It is the virtuous cycle feature of LHSs, whereby research and evidence‐informed practice generates new knowledge that is, in turn, applied to lived experience leading to new research and knowledge, that connects our commitment to health equity to the MCBK initiative [15]. Its goals are articulated in the initiative's 2018 Manifesto:

Knowledge has the potential to improve healthcare, the health of individuals, and the health of populations. Every decision affecting health should be informed by the best available knowledge. For moral and ethical reasons, it is imperative that each and every member of society has access to what is known at the time they are making health‐related choices and decisions [16].

The MCBK‐NA HEAL Workgroup, made up almost exclusively of health librarians, “aims to fuse the spirit of the library community with the vision of MCBK in order to ensure that the health‐advancing promise of LHSs is driven by diverse stakeholders and rendered equitably accessible to all.” This aim is informed by the awareness that, “historically, libraries have served as societal repositories of knowledge; transdisciplinary library science has organized and rendered such knowledge accessible and actionable, empowering individuals and engendering learning communities” [17].

According to a 2022 report prepared by Foley on behalf of the former Health Education England mapping NHS Knowledge and Library Services onto a LHS framework, “A Learning Health System captures data from practice, generates knowledge from the data and puts the knowledge back into practice to improve care… This requires capabilities in digital (data and technology), knowledge management and quality improvement.” In England, integrated national and local Knowledge and Library Services are at the heart of knowledge management within the NHS, a “position that can be leveraged to support local, regional and national Learning Health Systems.” Significantly, ^“^By helping staff, learners and patients to use the right knowledge, at the right time, to enable better decision‐making, Knowledge and Library Services help to realize many of the commonly cited objectives of an LHS” [18].

The national NHS Knowledge and Library Services team, part of NHS England, leads the implementation of the Knowledge for Healthcare strategy. The foci are on mobilizing evidence, supporting service improvements, improving outcomes and enhancing the research enterprise. Commitment to “equity of access and opportunity” is central to this strategic approach. Overcoming a ‘postcode lottery’ formerly experienced by healthcare staff, this strategic approach has been successful in achieving equitable access to knowledge resources and services for all NHS staff and learners, enabling them to use both evidence from research and from wider information sources [4].

Librarians contribute their expertise by enabling the use of best evidence at the point of need, enhancing the skills of health professionals in finding and using knowledge, and working to maximize the value of investment in information resources. Taken as a whole, these supports are instrumental to the success of healthcare organizations and the development of LHSs. In supporting systems, librarians “can help organisations to achieve better outcomes for patients, improve value, reduce variation, improve knowledge generation, more effectively apply existing knowledge and boost clinical performance” [18].

Equity is equally an important value when working with information producers and suppliers. Health information literacy is also a crucial consideration; both at national and local levels, NHS librarians partner with librarians in other organizations to improve the information skills of health information consumers. Equity is also central to the work of health library teams as they support hospital trusts to provide reliable and accessible patient information [4].

We believe the example of the NHSs Knowledge and Library Services' approach to equity/inequity, which inherently supports the concept of an LHS, can help inform how we approach opportunities to improve health equity internationally. In this, we are aided and informed by the development of a larger MCBK Global community of interest, albeit with a primary focus on computable biomedical knowledge [3]. In 2022 during the 5th annual public MCBK meeting, the first “manifestly” global meeting of the MCBK community, librarians attending from the US and UK connected—grounded in a joint interest in how to address issues of equity, whether in regards to access to knowledge or in the creation of that knowledge. That partnership revealed to us that our work was akin to the proverbial “hand in glove,” where there is mutual benefit when multiple parties with their diverse approaches and perspectives come together with a focus on a single goal—in our instance, addressing the health equity/inequity challenge within systems. This in turn informed our quest to better understand how practitioners, researchers, and theorists from the biomedical and health information communities globally looked at the challenge and opportunities of health equity and LHSs.

This Special Issue is indebted to the work of *LHS* Editor‐in‐Chief, Charles Friedman, MD, who published a thought piece in the journal in 2025 [19] where he describes ten core values of LHSs, which create an environment where health equity can flourish. Several of the values, such as focusing on individuals, inclusiveness, transparency, accessibility, adaptability, having cooperative and participatory leadership, and scientific integrity, promote a fertile environment for championing health equity for all. In this issue, we see many views on how health equity is being championed in LHSs.

Williamson et al. offer an overview of how health equity is conceptualized in the LHS literature from 2007 to June 2024. Entitled “Learning Health System, and Bounded Justice: A Critical Scoping Review,” this article distills the literature into four interrelated themes. The concept of bounded justice focuses attention on overarching structural and systemic forces challenging health equity and seeks to understand and disrupt the socio‐historical conditions that keep societies from pursuing health equity. The literature covered in this scoping review illustrates that there is not one “right” way to embed equity into the science of LHSs.

Aberjirinde and Wood provide a philosophical approach by first observing three tensions that result from centering equity in LHSs: (1) divergent definitions and languages of health equity that they call “the tension of lexicon”, (2) rapid learning versus slow engagement that they call “the tension of pace”, and (3) equity as a driver and an outcome that they call “the tension of dual roles” in their article entitled “Navigating and Reframing Tensions Within Equity‐centered Learning Health Systems.” Under the tension of lexicon the authors state: “the definition is not as important as the principles and paradigms underpinning it [health equity]”. They go on to offer six principles of an equity‐oriented LHS: (1) system disruption, (2) privilege and power, (3) upstream determinants, (4) proportionate universalism, (5) epistemic recognition, and (6) participatory governance and accountability.

Two articles discuss integrating community into the LHS. In “It Takes a Village: Evolving from Learning Health System to Learning Community for Health Equity” Kraft et al. describe Dartmouth Health's effort to engage their community through the Center for Advancing Rural Health Equity (CARHE). CARHE brings together “four pillars”—researchers, educators, clinicians, and community constituents—to improve health equity in rural Northern New England. CARHE was designed to evolve the LHS to become a learning community for health equity (LCHE) and this paper describes their context, structure, process, and lessons learned. Similarly, Towfighi, Lindgren and Orechwa in “The Southern California Healthcare Delivery Science Center: A Model for Aligning Patients, Healthcare Systems and Researchers to Develop, Test and Disseminate Innovations to Achieve Health Equity” describe efforts to enable the Southern California Healthcare Delivery Science Center to advance health equity as a LHS by aligning community with researchers and health systems through grants, education, team building and other assistance.

Two articles discuss specific tools or architectures to integrate health equity into LHSs. Hudson et al. argue that accurately capturing race and ethnicity data in a LHS is an essential antecedent to health equity assessment and development. They propose a knowledge architecture model for LHSs that is intentionally designed to acquire information regarding race and ethnicity to its fullest dimensions as supplied by patients and families in “Knowledge Architecture for Race and Ethnic Group Defining in Learning Health Systems.” They advocate for patient and community engagement studios to facilitate patient/researcher partnerships in the scientific method.

In another article, Collaborative Quality Initiatives (CQI) are multi‐institutional LHSs that improve healthcare by optimizing processes, enhancing patient outcomes, and reducing costs. However, opportunities to address health inequities have gone largely unaddressed by CQIs. In “Equity Dashboards in a Statewide Surgical Quality Collaborative: A Mixed Methods Pre‐Implementation Study,” Isenberg et al. introduced an equity dashboard to a statewide surgical CQI and assessed user interpretability and utility of this dashboard.

The push for equity as a key feature of LHSs resonates across the MCBK community as evidenced by international contributions to the Issue. In “Enhancing Learning Health Systems in Primary and Community Care: Contributions of NHS Library and Knowledge Specialists,” Day and Goswami demonstrate that knowledge specialists use their expertise and skills to foster a learning culture within community healthcare settings. The authors describe three proof‐of‐concept studies, which explore:

- The value of placing an embedded knowledge specialist alongside primary healthcare professionals
- Discovery work about partnership approaches for delivering knowledge and library services within integrated care systems, and
- Work in partnership with public libraries helping citizens to navigate digital health so they can be informed partners in LHSs.

The three initiatives demonstrate that NHS library and knowledge specialists act as intermediaries within LHS teams to accelerate knowledge into practice, enabling LHSs to thrive. They provide a vast network of high‐quality digital resources to all staff and learners in the NHS, working across boundaries, facilitating knowledge transfer and underpinning LHSs at the community level.

Laws and Cockburn's “Data Fit for Health Equity: Learning Health Systems, AI and the STANDING Together Recommendations” presents Standards for Data Diversity, Inclusivity, and Generalizability or STANDING Together recommendations as a global best practice that LHSs can adopt when using AI tools to deliver care. STANDING Together includes a set of concepts to document health data sets (metadata) and recommendations for use of health datasets. Several national agencies and funders have acknowledged these recommendations, yet more needs to be done to ensure adoption.

The work of HEAL, the Knowledge and Library Services component of the NHS, MCBK‐NA, and MCBK Global is ongoing as is the commitment to health equity. We encourage members of the library and information sciences community who share a commitment to health equity to engage in this work that we believe is of mutual benefit to their professional community and the larger health care enterprise.

## Data Availability

Data sharing not applicable to this article as no datasets were generated or analyzed during the current study.
